# Case Report: Three-Dimensional Printing–Assisted Surgical Treatment of Complex Body Vein Ectopic Drainage

**DOI:** 10.3389/fcvm.2022.782601

**Published:** 2022-01-31

**Authors:** Luyao Wei, Guowen Sun, Qin Wu, Weizhi Zhang, Mi Tang, Tianli Zhao, Shijun Hu

**Affiliations:** ^1^Department of Cardiothoracic Surgery, The Second Xiangya Hospital, Central South University, Changsha, China; ^2^Department of Cardiothoracic Surgery, Chenzhou No. 1 People's Hospital, Chenzhou, China; ^3^Department of Echocardiography, The Second Xiangya Hospital, Central South University, Changsha, China

**Keywords:** complex body vein ectopic drainage, 3D reconstruction, 3D printing, inferior vena cava, hepatic vein, intubation complex body vein ectopic drainage, intubation

## Abstract

**Introduction:**

Complex ectopic drainage of body veins is a rare congenital disease. Its preoperative diagnosis and surgical choice can be considerable challenges.

**Case Summary:**

A 5-year-old patient was diagnosed precisely by preoperative transthoracic echocardiography, computed tomography (CT), three-dimensional (3D) reconstruction, and three-dimensional (3D) printing of the heart and great blood vessels. The operation was performed successfully using flexible intraoperative intubation strategies.

**Conclusion:**

3D printing technology can assist in the formulation of surgical protocols for complex body vein ectopic drainage. Flexible intubation strategies can increase the success of the operation.

## Introduction

Abnormal drainage of the inferior vena cava (IVC) into the left atrium (LA) is a rare congenital condition. Especially when associated with ectopic hepatic vein drainage and atrial septal defects, its preoperative diagnosis and surgical choice can be considerable challenges with no uniform standards (1, 2).

Here, we report a case of a patient with ectopic drainage of the inferior vena cava into the left atrium, ectopic drainage of the hepatic vein, and atrial septal defects. We precisely developed surgical protocol using 3D printing technology and successfully performed the operation of complex body vein ectopic drainage.

## Case Report

**Figure 1:**
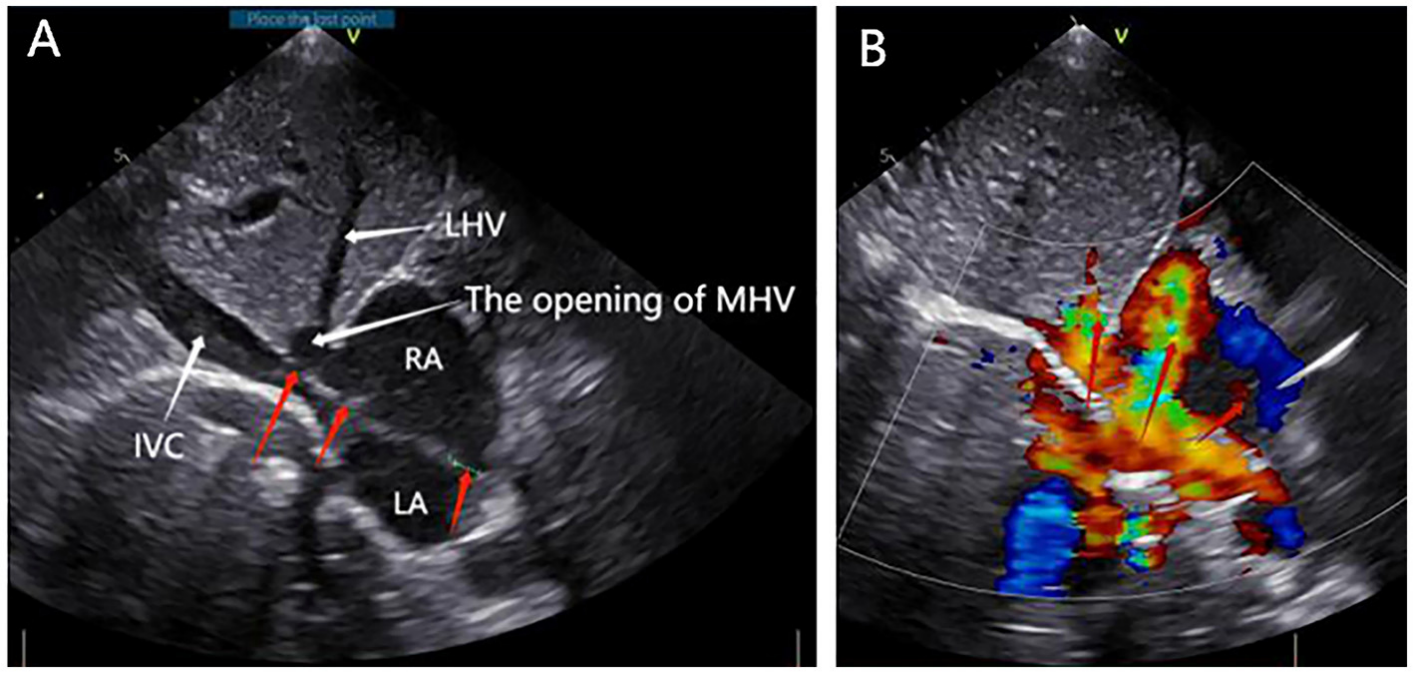
Preoperative transthoracic echocardiography. **(A)** Right atrium (RA), left atrium (LA), left hepatic vein (LHV), the opening of middle hepatic vein (MHV), inferior vena cava (IVC) (white arrowheads), and three atrial septal defects (ASD) (red arrowheads). **(B)** Blood flowed from the left atrium to the right atrium (red arrowheads).

**Figure 2:**
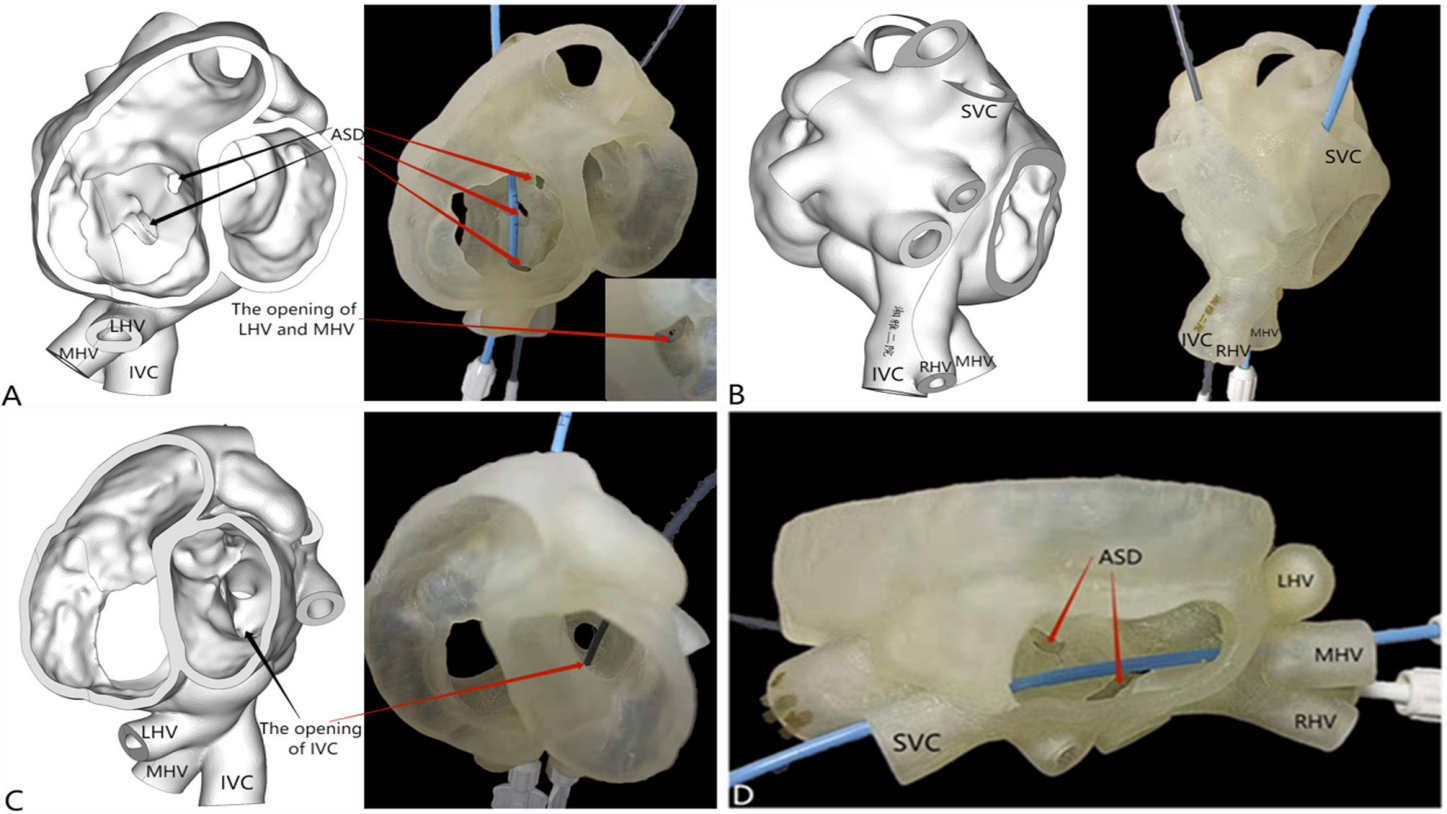
3D reconstruction and 3D printing. **(A)** Three large atrial septal defects (one of fossa ovalis type [diameter of 6 mm], one near the superior vena cava [diameter of 15 mm], and one near the inferior vena cava [diameter of 8 mm]); the left hepatic vein joined the middle hepatic vein, and the opening was located in the right atrium. **(B,C)** The right hepatic vein drained into the inferior vena cava, and the opening of the inferior vena cava was located in the left atrium. **(D)** Two atrial septal defects were visible from the right atrium incision level.

A 5-year-old boy with a history of allergy to milk and eggs experienced frequent colds. On physical examination, the patient was well-developed and showed no signs of hypoxia, such as cyanosis. He was found to have a grade 3/6 systolic ejection murmur between the second and third intercostal space along the left sternal border. Moreover, his blood oxygen saturation of the extremities was in the normal range. The patient underwent a detailed examination before the operation including chest radiography, electrocardiography, transthoracic echocardiography, computed tomography (CT), three-dimensional (3D) reconstruction and 3D printing of the heart and great blood vessels, and detailed laboratory examination. The patient's chest radiograph showed a slight swelling of the pulmonary artery. Transthoracic echocardiography showed three secondary atrial septal defects, and that a part of the right hepatic vein and inferior vena cava drained into the left atrium (Figures 1A,B). The 3D reconstruction and 3D printing models showed the following abnormalities: there were three atrial septal defects (one of fossa ovalis type, one near the superior vena cava, and one near the inferior vena cava); the left hepatic vein joined the middle hepatic vein, and the opening was located in the right atrium (Figure 2A); the right hepatic vein drained into the inferior vena cava, and the opening of the inferior vena cava was located in the left atrium (Figures 2B,C). Figure 2D shows that two atrial septal defects could be revealed from the right atrium incision level.

**Figure 3:**
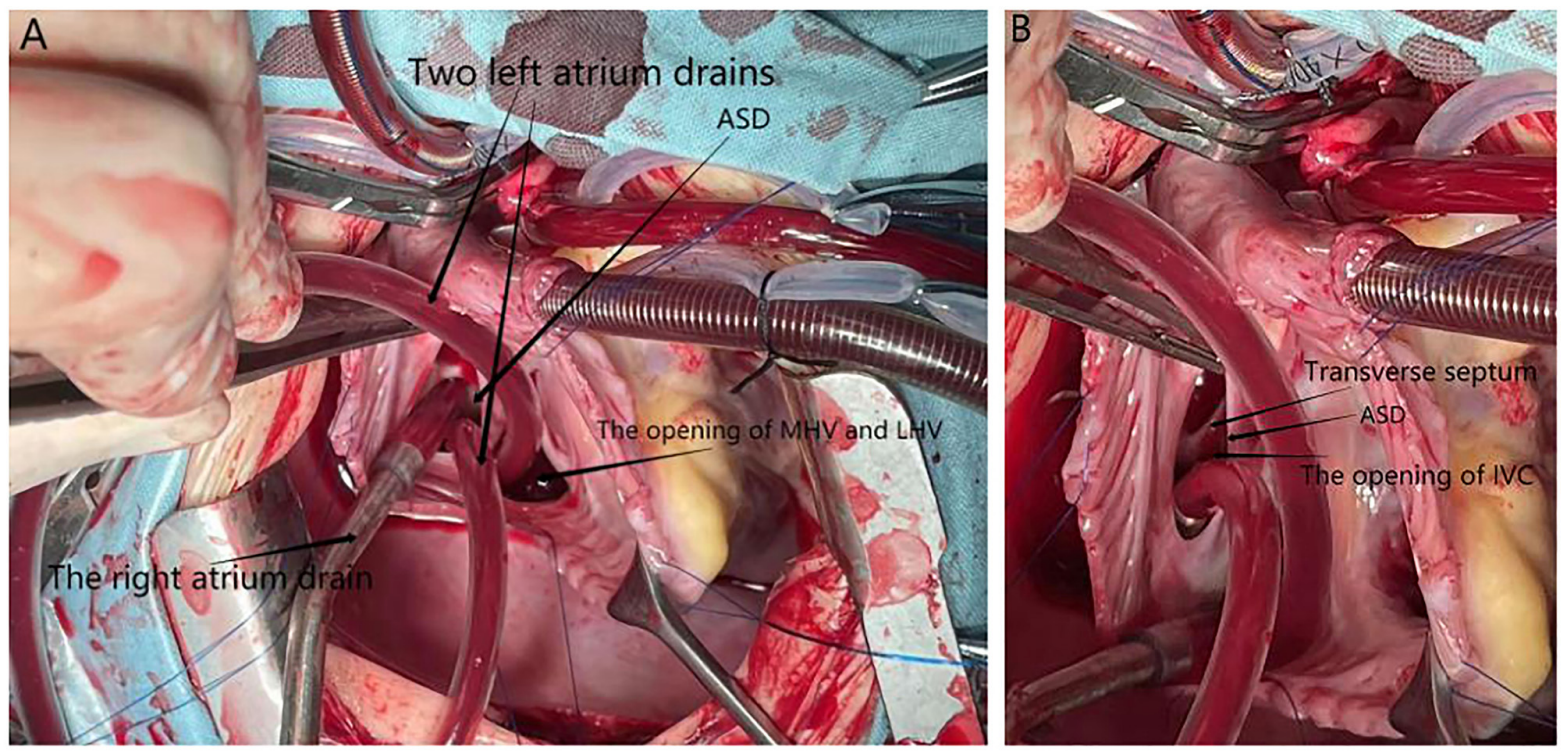
An intraoperative photograph (after the RA was cut off). **(A)** ASD, the opening of LHV and MHV, two left atrium drains, and one right atrium drain are indicated by black arrowheads. **(B)** Transverse septum at the orifice of the inferior vena in the left atrium and the opening of the inferior vena cava are indicated with black arrowheads.

During the establishment of cardiopulmonary bypass, routine aortic cannulation, superior vena cava cannulation, cold perfusion cannulation, and superior vena cava and aortic blockade were performed. Owing to the abnormality of the inferior vena cava opening, inferior vena cava cannulation was not performed. One right atrium drain and two left atrium drains were placed in the opening of the hepatic vein in the right atrium and the opening of the inferior vena cava in the left atrium through the atrial septal defect, respectively, for intracardiac blood drainage. The field was well-exposed, and the patient's blood perfusion and vital signs were good. The foramen ovale atrial septal defect was viewed directly, and the diameter was about 15 mm (Figure 3). The left hepatic vein joined the middle hepatic vein, and the opening was located in the right atrium (Figure 3A). During the operation, the interatrial septum was cut off, and we found the right hepatic vein draining into the inferior vena cava and the opening of the inferior vena cava in the left atrium next to the right inferior pulmonary vein (Figure 3B). A transverse septum, where the inferior vena cava met the left atrium, was found (Figure 3B). The atrial septal defect was closed by continuous suture of the bovine pericardium slice of the corresponding area with 5-0 prolene suture, and the openings of the hepatic vein and inferior vena cava were separated into the right atrium. The surgery was successful, and the patient was discharged from hospital on the fifth postoperative day. A postoperative echocardiogram performed on the third postoperative day demonstrated that there was no residual atrial shunt, and the blood supply to the heart from the IVC and the hepatic veins was corrected. At the 1-month follow-up, the patient was doing well.

## Discussion

To our knowledge, there have been several cases of patients with inferior vena cava complicated with pulmonary venous drainage and atrial septal defect (1–3). This is the first case describing the detailed clinical features, preoperative examination data, and surgical data of a patient with inferior vena cava complicated with abnormal hepatic venous drainage and atrial septal defect. Our experience with our first case cautions surgeons who may encounter patients with ectopic drainage of inferior vena cava and hepatic vein.

Ectopic drainage of the inferior vena cava into the left atrium is usually associated with cyanosis (1–4). However, this patient did not have obvious signs of cyanosis and had good blood oxygen saturation of the extremities, which may be due to the large diameter of the atrial septal defect and the role of the transverse septum at the orifice of the inferior vena cava in the left atrium. Because the pressure in the LA is higher than that in the RA in the early stage of the disease, the septum carries most of the blood from the IVC into the RA through the ASD, and oxygenated blood in the pulmonary circulation is transported to the whole body. As the disease progresses, significant left-to-right shunting tends to lead to pulmonary hypertension. Increased pulmonary artery pressure would reduce left-to-right shunt; therefore, a large amount of blood from IVC would flow to the systemic circulation, thereby increasing symptoms of hypoxia such as decreased oxygen saturation. Thus, the patient needed treatment to prevent further progression of the disease.

On the basis of preoperative transthoracic echocardiography and CT examination of cardiovascular blood vessels accompanied with 3D reconstruction and 3D printing, we were able to visualize the patient's cardiac abnormalities well, laying a good foundation for the choice of surgical methods. CT imaging has many advantages in diagnosing and treating cardiovascular diseases, with clearer images and faster scanning speed. 3D printing models can reflect complex cardiovascular malformations more intuitively and accurately (5). We clearly observed the abnormal drainage of the inferior vena cava and hepatic veins through 3D reconstruction and 3D printing model. 3D imaging aids in surgical planning and reduces redundant surgical interventions. In addition, it may also help to reduce the rate of misdiagnosis and surgical errors (6).

Since the inferior vena cava cannulation was not performed during the establishment of cardiopulmonary bypass, the three atrium drains placed in the left atrium and the right atrium fully exposed the surgical field of view and facilitated the operation, indicating that cannulation and occlusion of the inferior vena cava do not need to be performed when there is a way to drain the blood from the inferior vena cava during the establishment of cardiopulmonary bypass to guide operations in the future.

## Conclusion

This is the first case of the ectopic drainage of the inferior vena cava into the left atrium with ectopic drainage of the hepatic vein and atrial septal defects. 3D printing technology can assist in the formulation of surgical protocols for complex body vein ectopic drainage. Flexible intubation strategies can increase the success of the operation.

## Data Availability Statement

The original contributions presented in the study are included in the article/supplementary material, further inquiries can be directed to the corresponding author/s.

## Ethics Statement

Ethical review and approval was not required for the study on human participants in accordance with the local legislation and institutional requirements. Written informed consent to participate in this study was provided by the participant's legal guardian/next of kin. Written informed consent was obtained from the minor's legal guardian/next of kin, for the publication of any potentially identifiable images or data included in this article.

## Author Contributions

TZ, QW, GS, and SH were responsible for the diagnosis and treatment of the patient. LW collected clinical data and prepared the manuscript. All the authors have read and approved the final manuscript, have agreed to be accountable for the content of the work, contributed to the article, and approved the submitted version.

## Funding

This study was supported by the Changsha Natural Science Foundation, Hunan province, China (kq2014231).

## Conflict of Interest

The authors declare that the research was conducted in the absence of any commercial or financial relationships that could be construed as a potential conflict of interest.

## Publisher's Note

All claims expressed in this article are solely those of the authors and do not necessarily represent those of their affiliated organizations, or those of the publisher, the editors and the reviewers. Any product that may be evaluated in this article, or claim that may be made by its manufacturer, is not guaranteed or endorsed by the publisher.
